# Mechanism of Ferroptosis: A Potential Target for Cardiovascular Diseases Treatment

**DOI:** 10.14336/AD.2020.0323

**Published:** 2021-02-01

**Authors:** Jie Ju, Ya-nan Song, Kun Wang

**Affiliations:** ^1^Institute of Translational Medicine, The Affiliated Hospital of Qingdao University, College of Medicine, Qingdao University, China.; ^2^Medical College of Qingdao University, Qingdao, China.

**Keywords:** ferroptosis, reactive oxygen species, iron, cardiovascular diseases

## Abstract

Ferroptosis is a form of programmed cell death caused by production of reactive oxygen species and disequilibrium of iron homeostasis. Many chemical compounds and clinical drugs induce ferroptosis in normal and cancer cells, while peroxidation inhibitors, iron chelators, and antioxidants can block ferroptosis. Glutathione peroxidase 4, ferroptosis suppressor protein 1, nuclear factor erythroid 2-related factor 2, and system Xc^-^ are the negative regulators of ferroptosis, whereas nicotinamide adenine dinucleotide phosphate oxidase, p53, mitochondria voltage-dependent anion channel, and cysteinyl-tRNA synthetase function as positive regulators. Ferroptosis plays important roles in pathogen infection and tumor immunology. Recent studies suggest that ferroptosis plays a vital role in the pathogenesis of cardiovascular diseases (CVDs), which seriously threaten human health. Potential therapies designed around ferroptosis may alter the pathological progression of CVDs. Therefore, we redacted an overview of the discovery of ferroptosis, its regulatory mechanisms, and its potential impact on CVDs treatment.

## 1. Introduction

**Figure 1.**
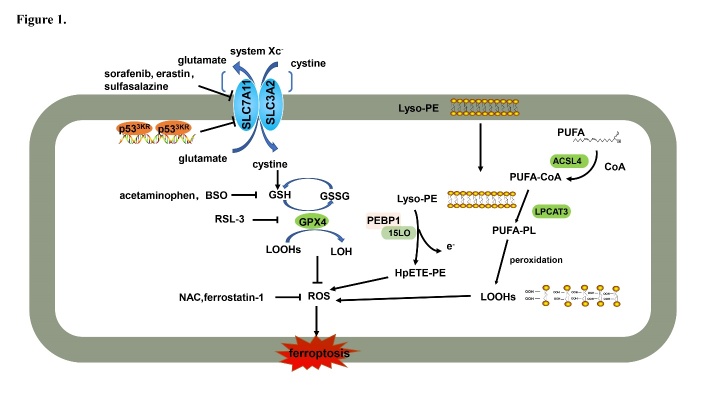
GPX4 is a key modulator of ferroptosis. Ferroptosis is induced by class I inducers (e.g., Erastin, BSO) and class II inducers (e.g., RSL-3), which inhibit GPX4 activity indirectly or directly, respectively. GPX4 is an inhibitory protein of lipid ROS production that catalyzes the reduction of lipid hydroperoxides. BSO: buthionine sulfoximine; PEBP1: phosphatidylethanolamine-binding protein 1; 15LO: 15-lipoxygenases; Lyso-PE: lysophosphatidylethanolamine; HpETE-PE: hydroperoxy eicasotetraenoyl phosphatidylethanolamine; PUFA: polyunsaturated fatty acid; CoA: coenzyme A; PL: lysophosphatide; GSSG: oxidized glutathione; GSH: reduced glutathione; ACSL4: acyl-CoA synthetase long-chain family member 4; LPCAT3: lysophosphatidylcholine acyltransferase 3; LOOH: polyunsaturated fatty acid hydroperoxides; LOH: hydroxy lipid species; GPX4: glutathione peroxidase 4; NAC: N-acetyl-l-cysteine; ROS: reactive oxygen species; SLC7A11: solute carrier family 7 member 11; SLC3A2: solute carrier family 3 member 2.

**Figure 2.**
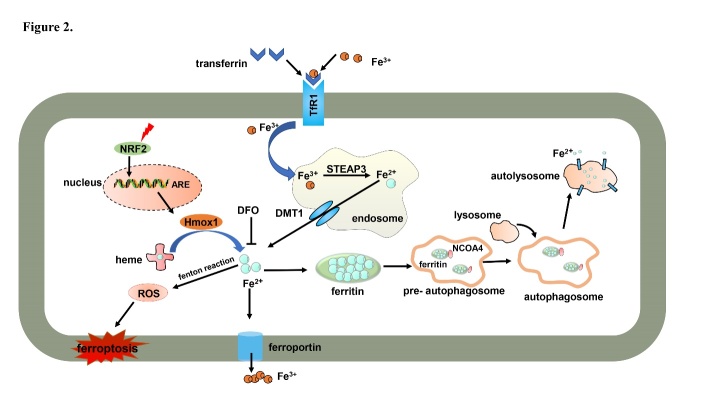
Iron metabolism of ferroptosis. Intracellular iron is imported and exported outside the cells by ferroportin. Iron is stored by ferritin, which can be degraded by NCOA4-mediated ferritinophagy. In addition, Hmox1 catalyzes heme degradation and releases iron. Excess iron induces ROS production by the Fenton reaction to initiate ferroptosis. NRF2: erythroid 2-related factor 2; Hmox1: heme oxygenase-1; ROS: reactive oxygen species; DFO: deferoxamine; DMT1: divalent metal transporter 1; STEAP3: STEAP family member 3; TfR1: transferrin receptor 1; NCOA4: nuclear receptor coactivator 4; NRF2: erythroid 2-related factor 2; Hmox1: heme oxygenase-1; ROS: reactive oxygen species; DFO: deferoxamine; DMT1: divalent metal transporter 1; STEAP3: STEAP family member 3; TfR1: transferrin receptor 1.

**Figure 3.**
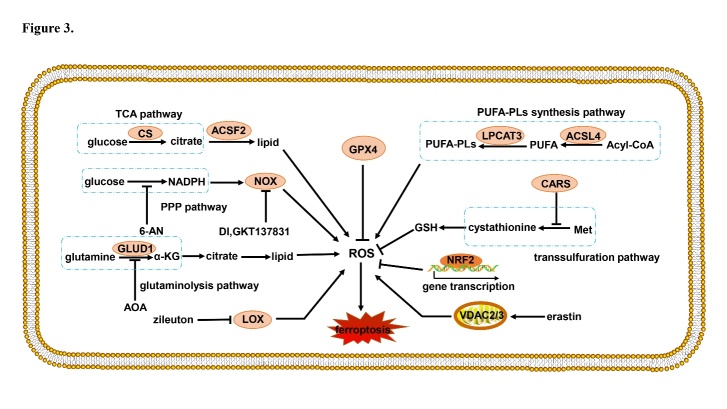
ROS production mediated by diverse signaling pathways. LOX, VDAC2/3, TCA, PPP, glutaminolysis, and lipid synthesis pathways promote ROS production. Antioxidative genes regulated by NRF2 and reductive GSH inhibit ROS production. CS: citrate synthase; ACSF2: acyl-CoA synthetase family member 2; NOX: nicotinamide adenine dinucleotide phosphate oxidase; NADPH: nicotinamide adenine dinucleotide phosphate; PPP: pentose phosphate pathway; GLUD1: glutamate dehydrogenase 1; AOA: amino-oxyacetate; α-KG: α-ketoglutarate; LOX: lipoxygenases; GPX4: lipoxygenases; ROS: reactive oxygen species; GSH: reduced glutathione; NRF2: erythroid 2-related factor 2; VDAC: mitochondrial voltage-dependent anion channel; Met: methionine; CARS: cysteinyl-tRNA synthetase; LPCAT3: lysophosphatidylcholine acyltransferase 3; ACSL4: acyl-CoA synthetase long-chain family member 4; 6-AN: 6-aminonicotinamde; PUFA: polyunsaturated fatty acid.

Cell death plays important roles throughout all life stages, both in the embryo development and in growth, maturation, and senescence processes: it is vital for various aspects of health, such as homeostasis, pathology, and other biological responses. Ferroptosis is a recently recognized form of cell death that has attracted considerable attention in explaining the molecular processes that control the demise of cells. The core of ferroptosis is the selenoenzyme glutathione peroxidase 4 (GPX4), which contains the rare amino acid selenocysteine at its active site, which catalyzes the reduction of polyunsaturated lipid hydroperoxides (LOOHs) species to non-toxic hydroxy lipid species, thus preventing uncontrolled peroxidation of polyunsaturated fatty acids (PUFAs) and PUFA-containing membrane phospholipids (Fig. 1) [1]: oxidized lipids disrupt the barrier function of biological membranes by forming hydrophilic pores. Normal levels of iron are essential for growth and proliferation. However, the interaction between LOOHs and excessive ferrous iron (Fe^2+^) generates reactive oxygen species (ROS) that amplify lipid peroxidation reactions and generate highly reactive products detrimental to DNA or proteins [2]. In summary, iron imbalance (Fig. 2) and ROS production (Fig. 3), mainly caused by lipid peroxidation, are the key hallmarks of ferroptosis.

The heart is one of the busiest organs and the hardest working muscle in the body. The role of the heart is to pump blood, thus supplying oxygen and nutrition to organs and tissues and removing the end products of metabolism [3]. Currently, cardiovascular diseases (CVDs) have become the world's leading killer being responsible for 40% of all deaths (41 million people per year), so they seriously impact on human health. The World Health Organization reported in 2014 that the mortality of cardiovascular diseases should be reduced by 25% [4]. Therefore, studying the pathogenesis of cardiovascular diseases, especially cardiopathy, and finding viable therapeutic targets are problems requiring a prompt solution.

Studies have revealed that iron homeostasis imbalance, oxidative stress, inflammatory response, energy metabolism disorder, and mitochondrial damage come into play in cardiovascular diseases. This paper will mainly describe the mechanism of ferroptosis and the research progress in cardiovascular diseases, providing new ideas for the treatment of cardiovascular diseases.

## 2. Discovery of ferroptosis

In 1959, Harry Eagle tried to detect which substance were essential for metabolism; he found that, out of 13 different amino acids, only cysteine deficiency inhibits the growth of human and mouse cells. Cells cultured in a cysteine-deficient medium show a distinct morphology, different from other amino acids deprivation [5]. In the following years, studies have confirmed that cysteine deficiency and glutathione synthesis inhibition are important causes of cell death [6-8]. Only recently they were officially recognized as important characteristics of ferroptosis [2, 7], while other studies have found that both iron chelators and lipophilic antioxidants prevent the occurrence of this kind of cell death.

By screening small molecule pools to identify compounds selectively lethal for cells expressing the mutant oncogene HRAS, the Stockwell Laboratory identified in 2003 a compound named “erastin” [9]. The direct target of erastin was identified as a Na^+^ independent cystine/glutamate antiporter called system Xc^-^; their interaction would block cystine uptake and then induce ferroptosis [9]. Only in 2012, ferroptosis was named for the first time and described as a regulated cell death dependent on excessive iron, and was characterized by the excessive presence of ROS and redox active iron [10]. A second ferroptosis inducer, the diastereoisomer 1S,3R-RSL3 (RSL3), was identified as a GPX4 inhibitor. RSL3 covalently binds and inactivates GPX4 to induce intense and selective lethality in HRAS^V12^-expressing BJeLR-derived cell lines but not in wild-type HRAS cell lines [1, 2, 11].

## 3. Morphology of ferroptosis

**Figure 4.**
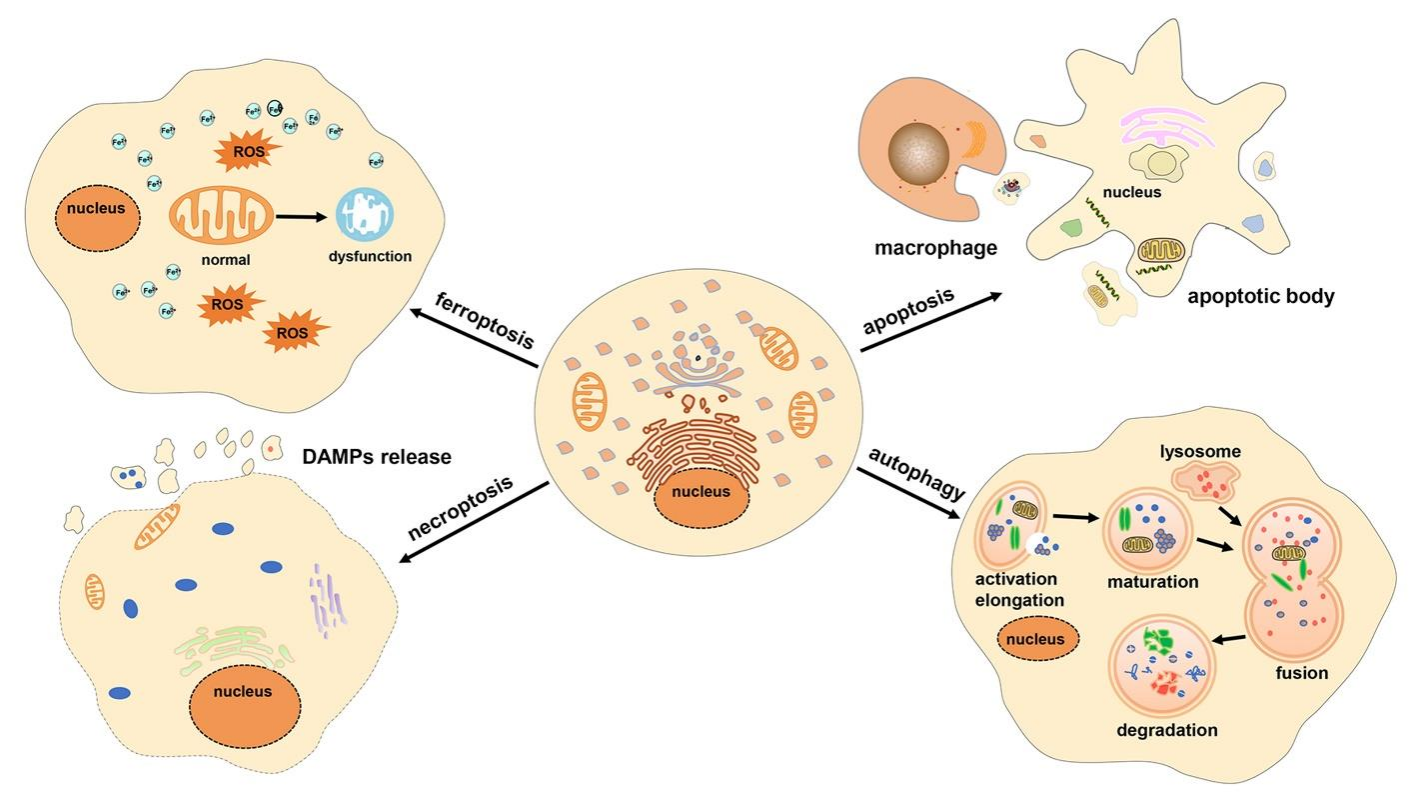
Distinctive forms of programmed cell death and their morphological characteristics. Ferroptosis does not result in chromatin condensation, loss of plasma membrane integrity, or the formation of double membrane-layered autophagosomes. The most evident morphology of ferroptosis is the smaller than normal mitochondria, with increased membrane density and no cristae. In addition, the metabolic disturbance of iron and excess ROS production are the main characteristics of ferroptosis. Apoptosis is characterized by cell shrinkage, membrane blebbing, chromatin condensation, and formation of apoptotic bodies, which are then devoured by neighboring macrophages. Necroptosis occurs as a consequence of irreparable cell damage and is characterized by cell swelling and plasma membrane rupture. Cellular contents that are released outside the cell cause an inflammatory response in the surrounding tissue. Autophagy is characterized by the formation of double-membrane vesicles called autophagosomes. The fusion of autophagosomes and lysosomes forms autolysosomes, and their inclusions are then digested by hydrolases. ROS: reactive oxygen species; DAMPs: damage-associated molecular patterns.

Ferroptosis is mainly characterized morphologically by shrunken mitochondria, which are smaller than normal mitochondria and primarily exhibit increased membrane density as well as cristae degeneration and breakdown, with few other significant morphological changes before ferroptosis [12]. Ultrastructural analysis demonstrated that ferroptosis involves outer mitochondrial membrane rupture in immortal mouse embryonic fibroblasts (MEFs) [13]. In addition, the morphology of the nucleus remains unchanged, not showing chromatin condensation. On the contrary, margination, condensation, and fragmentation of chromatin are typical features of classical apoptosis [2]. Excessive accumulation of ROS causes membrane lipid peroxidation, which damages the barrier function of the cell membrane. In addition, generation of apoptotic bodies and plasma membrane blebbing are other features of apoptosis. Necroptosis involves cell swelling, rupture of the cell membrane, and formation of annexin V-positive membrane bubbles, which relies on the mixed lineage kinase domain-like pseudokinase activation and on the endosomal sorting complexes required for transport-III [14]. None of these processes are present during ferroptosis and therefore distinguish ferroptosis from the other two described forms of regulated cell death. Another form of regulated cell death is autophagy, which is characterized by *de novo* synthesis of double-membrane vesicles loaded with organelles such as mitochondria; successive fusion with a lysosome leads to their digestion. This representative morphology of autophagy is also not found during ferroptosis [15]. Ferroptosis is thus morphologically distinguishable from apoptosis, necroptosis, and autophagy; therefore, we compared the four different cell death patterns by four aspects, as shown in Table 1, and in the class pattern diagram shown of Fig. 4 [16-20].

### 3.1. The ferroptosis pathway

Iron metabolism disorder (Fig. 2) and ROS production (Fig. 3) are key events in the ferroptosis cascade[10, 21] and as such need to be examined singularly.

#### 3.1.1 Iron metabolism disorder

Ferroptosis is mediated by intercellular excessive iron; when coupled with transferrin, extracellular ferric iron (Fe^3+^) is imported into cells through the membrane protein transferrin receptor 1 (TfR1). Within the cell, Fe^3+^ is located in the endosome and is then reduced to Fe^2+^ by the metalloreductase STEAP family member 3 [22]. Fe^2+^ is released into the cytoplasm iron pool through the mediation of the divalent metal transporter 1 [23]. Cellular iron can be oxidized from Fe^2+^ to Fe^3+^ and exported by ferroportin to maintain normal iron levels. Alternatively, excess iron is stored in ferritin and forms redox-inactive ferritin heteropolymers to preserve tissues and cells against ferroptosis-mediated injury. In particular circumstances, the nuclear receptor coactivator 4 (NCOA4)-mediated ferritin degradation increases iron levels to induce ferroptosis [12, 24, 25]. When treating HRAS^V12^ mutant fibrosarcoma cells with erastin, TfR1 mRNA and protein levels gradually increase, but the expression level of both ferritin heavy chain 1 (FTH1) and ferritin light chain decreases. This suggests that increased iron uptake via TfR1 upregulation and reduced iron storage by FTH1 and ferritin light chain downregulation contribute to iron overload during ferroptosis [11]. Upregulation of heme oxygenase-1 (Hmox1) promotes heme degradation and iron release in mouse heart tissue upon administration of doxorubicin (DOX) (Fig. 2) [26]. In addition, heat shock protein family B member 1 phosphorylation mediated by protein kinase C negatively regulates iron-mediated lipid ROS production in erastin-treated HeLa cells [27]. Mutation of some iron metabolism-related genes such as homeostatic iron regulator, hemojuvelin, causes hemochromatosis, an iron-overloading disease. Both hemojuvelin and hepatocyte specific Smad4 gene knockout mice develop high iron overload, which leads to hepatic ferroptosis. On the contrary, homeostatic iron regulator knockout mice develop only mild iron overload and do not show hepatic ferroptosis; furthermore, their liver damage is recovered by ferrostatin-1 [28].

NCOA4 maintains intracellular and systemic iron homeostasis by mediating the selective autophagic degradation of ferritin (process known as ferritinophagy). Inhibition of ferritinophagy or knockdown of NCOA4 inhibit the accumulation of reactive iron and ROS as well as the eventual development of ferroptosis [29]. NCOA4 interacts with HERC2 ubiquitin E3 ligase and mediates ubiquitin-dependent proteasome degradation when cellular iron levels are high [30]. NCOA4 combined with iron-loaded ferritin is delivered into the pre-autophagosome structure. Ferritin is degraded in the lysosome after fusion with the autophagosome. The iron is thus released into the lysosome by ferritin and then transferred in the cytoplasm. These free iron ions can participate in the biological synthesis of heme and in other physiological processes (Fig. 2) [31].

**Table 1:** Characteristics of different forms of cell death.

Type	Morphological features	Biochemical features	Activation manner	Regulated genes
Ferroptosis	Small mitochondria with increased mitochondrial membrane densities; Outer mitochondrial membrane rupture and normal nucleus	iron metabolism and ROS production	Erastin; Sorafenib; RSL3	Positive regulation: VDAC2/3; RAS; NOX; TfR1; p53; CARS; ACSL4; Hmox1; NCOA4 Negative regulation: GPX4; NRF2; HSPB1; SLC7A11; FSP1; PEBP1; 15LO
Apoptosis	Plasma membrane blebbing without rupture; chromatin condensation and nuclear fragmentation; formation of apoptotic bodies	Caspase activation; DNA fragment; PS exposure	Physiological or pathological conditions	Positive regulation: Caspase; Cyt c; BH3 family; Bax family; p53 Negative regulation: Bcl-2 family
Necroptosis	Rupture of plasma membrane; cytoplasmic swelling; organelles swelling; moderate chromatin condensation	RIPK1, RIPK3 and MLKL phosphorylation; ROS production; DAMPs release	TNF-α plus pan-Caspase inhibitor co-treatment; HSV-1 infection	Positive regulation: RIPK1; RIPK3; MLKL Negative regulation: Flotillin; syntenin-1
Autophagy	accumulation of double-membraned autophagic vesicle	LC3-I to LC3-II conversion; p62 degradation	Nutritional deficiencies; oxidative stress	Positive regulation: ATG5; ATG7; Beclin 1 Negative regulation: mTOR

#### 3.1.2 ROS production

One of the main inductors of ferroptosis is ROS presence, which is caused by nicotinamide adenine dinucleotide phosphate oxidase (NOX) action, transsulfuration pathway activities, and lipid or PUFA peroxidation. In addition, many proteins negatively regulate ROS production, such as GPX4 (Fig. 3). Treatment with erastin leads to the inactivation of GPX4, inducing mitochondrial dysfunction, an unbalance of cellular redox homeostasis, and generation of lipid peroxides, which altogether unleash ferroptosis [32, 33].

Mitochondrial fatty acid metabolism is important to trigger or moderate ferroptosis induced by cysteine deprivation. The main functions of mitochondria are the tricarboxylic acid (TCA) cycle and electron transport required for lipid ROS generation, which trigger ferroptosis. However, ferroptosis induced by GPX4 depletion is independent of mitochondrial functions [34]. The citrate synthase of the TCA cycle and the acyl-CoA synthetase family member 2 of the fatty acid synthesis pathway are key components of fatty acid metabolism: silencing acyl-CoA synthetase family member 2 and citrate synthase specifically attenuates erastin-induced ferroptosis [10].

Nicotinamide adenine dinucleotide phosphate is the main product of the pentose phosphate (PPP) pathway, which participates in lipid synthesis by providing reducing power. NOX generates ROS in various tissues as part of its normal physiological functions [35]; NOX inhibitors DI, GKT137831, and PPP pathway inhibitor 6-aminonicotinamide thus inhibit ferroptosis by attenuating ROS production [36].

Glutamate is an important molecule that induces ferroptosis, which cannot be commenced by cells without this amino acid. The destiny of glutamate is to be transformed into α-ketoglutarate by glutamate dehydrogenase 1-mediated deamination, or into a lipid by transaminase-mediated transamination. Treatment with the transaminase inhibitor amino-oxyacetate or by knockdown of glutamic-oxaloacetic transaminase 1 inhibits ferroptosis. Moreover, TCA cycle products of glucose also convert to lipids and participate in ROS production [10].

ROS react with PUFAs in lipid membranes and induce lipid peroxidation. Acyl-CoA synthetase long-chain family member 4 (ACSL4) is essential for ferroptosis. Specifically, GPX4 and ACSL4 double-knockout cells resist ferroptosis [37]. Nine genes involved in ferroptosis were identified in KBM7 cells by massive insertional mutagenesis; those included mediators of ACSL4 and lysophosphatidylcholine acyltransferase 3 (LPCAT3) (Fig. 1), but only a little protective effect was observed in LPCAT3 knockout Pfa1 cells [37, 38]. This indicates that ACSL4 plays a more widespread role in ferroptosis and that the function of LPCAT3 is restricted to certain cellular subtypes.

Arachidonic acid and linolenic acid are substrates of lipoxygenases (LOX). The LOX family plays a vital role in the synthesis of LOOHs, which participates in various pathologies including neurodegeneration, ischemia-reperfusion injury (IRI), and infection. LOX-overexpressing cells are inclined to lipid peroxidation and ROS production, which sensitize to ferroptosis induced by erastin or RSL3, in spite of slightly different extents [39].

**Table 2:** Genes involved in ferroptosis regulation.

Genes	Name	Function	Effect
NOX	NADPH oxidase activator	Produces ROS which participates in host defense, hormone biosynthesis, oxygen sensing and signal transduction.	Regulate ferroptosis Positively
TfR1	Transferrin receptor protein 1	Mediates iron uptake	Regulate ferroptosis Positively
ACSL4	Acyl-CoA synthetase long-chain family member 4	Converts free fatty acids into fatty acyl CoA	Regulate ferroptosis Positively
p53	Cellular tumor antigen p53	Inhibits SLC7A11 expression	Regulate ferroptosis Positively
CARS	cysteinyl-tRNA synthetase	Participate in trans-sulfuration pathway and synthesis of GSH	Regulate ferroptosis Positively
MAPK	Mitogen-activated protein kinase 1	Mediates cell growth, adhesion, survival and differentiation	Regulate ferroptosis Positively
NCOA4	Nuclear receptor coactivator 4	Mediates iron metabolism	Regulate ferroptosis positively
15LO	15-lipoxygenases	Catalyzes the formation of pro-ferroptotic 15-OOH-AA (HpETE)	Regulate ferroptosis positively
PEBP1	Phosphatidylethanolamine-binding protein 1	Inhibits the Raf/MEK/ERK cascade	Regulate ferroptosis positively
Hmox1	Heme oxygenase 1	Catalyzes degradation of heme	Regulate ferroptosis positively
GPX4	Glutathione peroxidase 4	Reduces phospholipid hydroperoxide	Regulate ferroptosis negatively
SLC7A11	Cystine/glutamate antiporter solute carrier family 7 member 11	Mediates cystine uptake and glutamate release	Regulate ferroptosis negatively
HSPB1	Heat shock protein beta-1	HSPB1 phosphorylation is negative regulated iron-mediated lipid ROS production	Regulate ferroptosis negatively
NRF2	Nuclear factor erythroid 2-related factor 2	Binds to ARE elements in the promoter regions of target genes	Regulate ferroptosis negatively
FSP1	ferroptosis suppressor protein	Reduces phospholipid hydroperoxide	Regulate ferroptosis negatively

### 3.2. Ferroptosis regulation

The ferroptosis process involves multiple proteins such as p53, small G-protein Ras (RAS), VDAC2/3, mitogen-activated protein kinase (MAPK), NCOA4, NOX, cysteinyl-tRNA synthetase (CARS), and others. Also, some negative regulators genes of ferroptosis exist, such as GPX4, system Xc^-^ and erythroid 2-related factor 2 (NRF2), which protect cells against ferroptosis; knockout of these genes induces ferroptosis *in vitro*, as shown in Table 2.

#### 3.2.1 Positive regulator

VDAC is a mitochondrial voltage-dependent, anion selective channel that facilitates transmembrane transport of ions and metabolites. Erastin is a VDAC-binding small molecule that possibly influences ion selectivity and allows cationic species into mitochondria selectively, which is lethal to some cancer cells [33]. Erastin-induced ferroptosis stops the interaction between VDAC and tubulin, thereby enhancing mitochondrial metabolism and limiting aerobic glycolysis in HepG2 cells [33, 40].

HRAS, NRAS, and KRAS belong to the small GTPase superfamily, which are mutated in approximately 30% of all cancers [41]. Erastin is lethal to HRAS mutant BJeLR cells, NRAS mutant HT-1080 cells, and KRAS-mutant Calu-1 cells [11]. Nevertheless, lymphocyte T cells, synchronized kidney tubular cells, and mouse embryonic fibroblast (MEF) cells are sensitive to erastin, independently of the RAS [42-44].

The transcription factor p53 is a crucial protein in multicellular organisms; the inactivation of the p53 tumor suppression pathway is inclined to transform most human tissue cells to cancer cells [45]. Recent studies have found that p53 directly binds to the flanking region of the human solute carrier family 7-member 11 (SLC7A11) gene, thus severely reducing SLC7A11 expression and activating ferroptosis [44]. Notably, p53^3KR^, an acetylation-defective p53 mutant that fails to induce cell cycle arrest, apoptosis, and senescence, still exhibits tumor suppression by binding the promoter of the SLC7A11 gene to active ferroptosis (Fig. 1) [44]. SLC7A11 mRNA expression is suppressed in p53^3KR/3KR^Mdm2^-/-^embryos, which induces ferroptosis to a large extent. P53^3KR/3KR^Mdm2^-/-^ embryos treated with ferrostatin-1 showed clear organogenesis after day E14.5. Overall, p53-mediated ferroptosis plays an important role in the defective embryonic development of p53^3KR/3KR^Mdm2^-/-^ mice [44].

The mitochondrial electron transport chain is a source of ROS in erastin-induced ferroptosis. NOX1, 2, 3, 4, and 5, and dual oxidase 1 and 2 are upregulated in several RAS mutant tumors [46]. Erastin-induced ferroptosis is strongly suppressed in human lung cancer Calu-1 cells by the canonical NOX inhibitor, the NOX1/4-specific inhibitor GKT137831, and by the PPP pathway inhibitor 6-AN, but also by shRNA-mediated silencing of two PPP enzymes, glucose-6-phosphate dehydrogenase and phosphoglycerate dehydrogenase [10, 36].

CARS (cysteinyl-tRNA synthetase) is the rate-limiting enzyme for cysteinyl-tRNA synthesis. Knockdown of CARS promotes transsulfuration pathway activation and serine biosynthesis, thus inhibiting erastin-induced cell death by preventing lipid ROS production, although it has no influence on iron homeostasis [47]. Moreover, repression of the transsulfuration pathway resensitizes cells to erastin. However, CARS knockdown cannot inhibit RSL3-induced lethality. The reason for this phenomenon is that RSL3 directly inhibits GPX4 biological activity; the inhibition is not avoided even increasing the levels of oxidized glutathione, reduced glutathione (GSH), or cysteine [47].

The MAPK family includes c-JUN N-terminal kinase (JNK), extracellular signal-regulated kinase (ERK), and p38. The ERK inhibitor, but not p38 or JNK inhibitors, inhibits the erastin-induced signal transducer and the activator of transcription 3 phosphorylation and ferroptosis in KRAS mutated PANC1 and CFPAC1 cells [48]. Knockdown of ERK1/2 and of upstream regulator MAPK kinase1/2 restrains the signal transducer and activator of transcription 3 phosphorylation and enhances resistance to erastin in PANC1 cells [48]. JNK and p38 inhibitors attenuate erastin-induced ferroptosis in human promyelocytic leukemia HL-60 cells. Together, these findings suggest that MAPK molecules involved in ferroptosis are cell-specific.

Phosphatidylethanolamine-binding protein 1 (PEBP1) is a small scaffolding protein that binds to Raf1 to repress its cascade reaction. Phosphorylated PEBP1 also binds to 15-lipoxygenases (15LO), which catalyzes the formation of pro-ferroptotic 15-OOH-arachidonic acid (HpETE) and esterifies phosphatidyl ethanolamine with HpETE to generate HpETE-PE [49]. The 15LO/PEBP1 complex stimulates PE oxygenation and forms 15-HpETE-PEs to induce ferroptosis. The disruption interactions between PEBP1 and Raf1 kinase liberates the protein and makes it available for binding 15LO to promote ferroptosis, which, in turn, can be blocked by GPX4 overexpression and ferrostatin-1 treatment (Fig. 1) [50].

#### 3.2.2 Negative regulator

GPX4 catalyzes the reduction of lipid peroxides in a complex cellular membrane environment to protect cells against oxidative damage [51]. Systemic deletion of GPX4, differently than the deletion of other members of the GPX family in mice, causes embryonic lethality, suggesting that GPX4 plays a key physiological role. The GPX4 inhibitor RSL3 induces ferroptosis dependently on excessive iron and ROS production without GSH depletion. GPX4 overexpression resists RSL3-induced ferroptosis [1]. Notably, liproxstatin-1 extends survival of acute renal failure in GPX4 conditional knockout mice compared with the vehicle-treated group [13]. GPX4 ablation results in membrane lipid peroxide accumulation and concomitant ferroptosis in lymphocyte T cells. CD8^+^ T cells originating from GPX4-deficient mice cannot maintain homeostatic balance in the peripheral immune system [43]. Moreover, GPX4 deficient CD4^+^ and CD8^+^ T cells infected by the leishmania parasite and acute lymphocytic choriomeningitis virus because of abnormal T cell expansion, can be rescued with treatment based on high doses of vitamin E [43].

System Xc^-^ is a member of the heterodimeric amino acid transporter family, consisting of a light chain (xCT, coded by the SLC7A11 gene) and a heavy chain (4F2hc, coded by the SLC3A2 gene) connected by disulfide bonds. Cystine is brought in the cytoplasm and exchanged with intracellular glutamate by system Xc^-^. Cystine is reduced to cysteine and is involved in GSH synthesis [52]. The anticancer activity of erastin improved by SLC7A11 depletion is consistent with the phenomenon that SLC7A11 overexpression diminishes erastin-induced anticancer activity [10]. Wang et al. found that deficiency of SLC7A11 increases susceptibility to iron overload and induces ferroptosis in hepatocytes and bone marrow-derived macrophages [28].

NRF2 plays a central role in protecting hepatocellular carcinoma cells against ferroptosis (Fig. 3) [53]. Knockdown of NRF2 and NRF2-targeted genes, glucose-6-phosphate dehydrogenase, and phosphoglycerate dehydrogenase, promotes erastin- or sorafenib-induced ferroptosis in hepatocellular carcinoma cells. Upregulated NRF2 protein promotes antioxidant protein expression (e.g., quinone oxidoreductase 1, Hmox1) and iron metabolism proteins (e.g., FTH1) in ferroptosis [53].

Recently, two research groups simultaneously reported that ferroptosis suppressor protein 1 (FSP1) is an effective ferroptosis-resistant factor screened in a CRISPR-Cas9 synthetic lethal pool and protects human cells from this type of cell death. FSP1 knockout cell lines are significantly more sensitive to ferroptosis inducers and are rescued by overexpressing FSP1. These findings reveal a previously unknown role for the lipid ubiquinone, which is found in lipid membranes, including mitochondrial ones, where it aids in the ATP production. Myristoylated FSP1 is located on membranes and lipid droplets, where FSP1 converts ubiquinone into ubiquinol, which inhibits peroxidation and blocks ferroptosis. Some tumor cells are susceptible to ferroptosis, and FSP1 mediates ferroptosis resistance in lung cancer cells and in mouse tumor xenografts. The discovery of FSP1 enhances the cognition of ferroptosis, which is critical for exploiting potential FSP1 inhibitors and ferroptosis-inducing drugs as strategies to overcome ferroptosis resistance in many cancers [54, 55].

### 3.3. Ferroptosis inducers

All ferroptosis-inducing agents can be classified into two categories based on whether they directly inhibit GPX4 activity. The first type of inhibitors, including erastin, sulfasalazine, diphenyleneiodonium chloride 2 (DPI2), buthionine sulfoximine (BSO), and lanperisone, blocks GPX4 activity through GSH depletion. The second type directly inhibits GPX4 without GSH depletion; this group includes RSL3 and the diphenyleneiodonium chloride (DPI) family, except for DPI2. The targets and functions of ferroptosis inducers are shown in Table 3.

Erastin induces cell death in two ways. First, erastin directly binds to mitochondrial VDAC2/3 in BJeLR cells accompanied by abnormal ROS generation by mitochondrial oxidation respiratory chain to induce ferroptosis [33]. Second, erastin inhibits system Xc^-^ activity and results in depletion of GSH, which inactivates GPX4 enzymes and then induces the formation of ROS, causing ferroptosis selectively in HRAS^V12^ mutant BJeLR cells [1, 10].

RSL3 binds and inactivates GPX4, which inhibits the peroxidase activity of GPX4 and increases lipid ROS levels to induce ferroptosis, but has no influence on the upstream of GPX4, such as GSH depletion and cysteine uptake.

BSO inhibits biosynthesis of GSH in the liver and other peripheral organs. BSO inhibits GPX4 through GSH depletion, which results in ferroptosis. Although BSO has the ability to induce ferroptosis, it is less potent than the system Xc- inhibitor erastin or GPX4 inhibitor RSL3. The reason why BSO only mildly induces ferroptosis is that inhibition of GSH synthesis upregulates a substitute antioxidant pathway to inhibit cell death [56].

**Table 3:** Ferroptosis inducers and inhibitors.

Reagents	Target	Function	Effect
erastin	System Xc^-^	Inhibits cystine import, causes GSH depletion	Induces ferroptosis
glutamate	System Xc^-^	High concentration glutamine inhibits cystine uptake and GSH synthesis	Induces ferroptosis
sorafenib	System Xc-	Blocks system Xc- activity and GSH synthesis	Induces ferroptosis
sulfasalazine	System Xc^-^	Low potency than erastin to induce ferroptosis	Induces ferroptosis
artemisnin	Unknown	Promotes iron-related genes expression	Induces ferroptosis
DPI2	GSH	Depletes GSH	Induces ferroptosis
RSL3	GPX4	Covalently binds to the GPX4 and promotes accumulation of lipid hydroperoxides	Induces ferroptosis
DPI7,10,12,13,17,18,19	GPX4	Covalently binds to the GPX4 and promotes accumulation of lipid hydroperoxides	Induces ferroptosis
Lanperisone	System Xc^-^	Promotes ROS production	Induces ferroptosis
Buthionine sulfoximine	GSH	Inhibits γ-glutamyl cysteine synthetase and GSH synthesis	Induces ferroptosis
Ferrostatin-1	ROS	Prevents ROS production from lipid peroxidation	Inhibits ferroptosis
Liproxstain-1	ROS	Prevents ROS production from lipid peroxidation	Inhibits ferroptosis
N-acetyl-l-cysteine	ROS	Scavenges cellular ROS	Inhibits ferroptosis
Vitamin E	5-Lipoxygenase	Maintains cellular redox homeostasis	Inhibits ferroptosis
Zileuton	5-Lipoxygenase	Maintains cellular redox homeostasis	Inhibits ferroptosis
SRS 11-92, SRS 16-86	ROS	Prevents ROS production from lipid peroxidation	Inhibits ferroptosis
Deferoxamine	Fe^2+^	Chelates excess iron	Inhibits ferroptosis
XJB-5-131, JP4-039	ROS	Scavenges cellular ROS	Inhibits ferroptosis
Mitoquinone	ROS	Inhibits mitochondrial superoxide generation	Inhibits ferroptosis

Lanperisone induces ferroptosis by promoting ROS production mediated by the RAS/Raf/MEK/ERK signaling pathway to kill KRAS-mutant mouse embryonic fibroblasts. However, lanperisone shows lower efficacy than erastin against KRAS-driven lung tumor growth [57].

Sulfasalazine is approved by the Food and Drug Administration for the treatment of chronic inflammation. Sulfasalazine inhibits SCL7A11 activity and induces ferroptosis in glioma cells at higher concentrations (>200μM) [58].

Sorafenib is a multikinase inhibitor approved in clinical settings as an anticancer drug for the treatment of hepatic carcinoma [59]. Sorafenib inhibits tumor cell proliferation by blocking the RAS/Raf/MEK/ERK signaling pathway, and attenuates tumor angiogenesis by blocking the vascular endothelial growth factor receptor 2 pathway and platelet-derived growth factor receptors [60]. Sorafenib induces ferroptosis similarly to erastin. The ferroptotic effect of sorafenib on hepatic carcinoma cells depends on the blockage of GSH synthesis rather than its inhibitory Raf signal pathway [60].

Artemisinin and its derivatives kill plasmodia parasites and cancer cells, depending on the presence of ferric iron ions. Artemisinin generates ROS and leads to oxidative stress in cancer cells, which contributes to cell death [61]. Artesunate is an artemisinin derivative that specifically induces ferroptosis in KRAS mutation pancreatic ductal adenocarcinoma cells but not in human pancreatic ductal epithelial cells.

All members of the DPI family can induce ferroptosis by inhibiting GPX4 activity. DPI2 follows the same mechanism as erastin to induce ferroptosis. BJeLR cells treated with DPI7, DPI10, DPI12, DPI13, DPI17, DPI18, and DPI19 cannot reduce phosphatidylcholine hydroperoxide, a specific substrate of GPX4, which indicates that these DPI family members directly inhibit GPX4 function [1].

### 3.4. Ferroptosis inhibitors

Ferroptosis induced by RSL3 or erastin can be blocked by most antioxidants (e.g., ferrostatin-1, liproxstatin-1, vitamin E), iron chelation (e.g., deferoxamine), and ROS scavengers (e.g., N-acetyl-l-cysteine, XJB-5-131, JP4-039, and Mitoquinone), as shown in Table 3 [1, 13, 21, 62-65].

Ferrostatin-1 and liproxstatin-1 attenuate the accumulation of lipid ROS by trapping radical antioxidants, but do not inhibit LOX activity and thus do not limit ferroptosis [63, 66]. Lipid cytomembrane constraining of ferrostatin-1 by its N-cyclohexyl moiety suppresses ferroptosis. The antioxidants vitamin E and α-tocotrienol are shown to inhibit ferroptosis via LOX suppression [67]. Zileuton, a LOX inhibitor, protects neurocytes from glutamate-induced oxidative injury by inhibiting ferroptosis [68].

Based on the chemical structure of ferrostatin-1, scientists have exploited the second-generation ferroptosis inhibitor SRS 11-92 and third-generation inhibitor SRS 16-86, which have higher potential for the treatment of renal IRI and promote functional recovery in contusion spinal cord injury [42, 69]. Liproxstatin-1 prevents ROS accumulation in GPX4^-/-^cells; this treatment also protects primary human renal proximal tubule epithelial cells from RSL3-induced cell death [10, 13]. Furthermore, daily intraperitoneal liproxstatin-1 administration remarkably extends survival compared with the vehicle-treated group mice and prevents tissue injury induced by renal IRI [13].

Superfluous iron produces ROS mediated by the Fenton reaction, which contributes to ferroptosis (Fig. 2). Thus, intracellular iron can be chelated by deferoxamine, which protects cells against ferroptosis induced by erastin treatment or GPX4 null-triggered cell death [22].

ROS is a characteristic component of ferroptosis and is mainly produced by lipid peroxidation. Supplementation with the ROS scavenger N-acetyl-l-cysteine significantly inhibits erastin-induced ferroptosis [1]. Recently, some ROS scavengers XJB-5-131 and JP4-039 were synthesized to inhibit ferroptosis by diminishing lipid ROS production [65]. Mitoquinone is a mitochondria-targeted ROS scavenger, which is sufficient against ferroptosis induced by erastin or GPX4 knockout, since during ferroptosis more lipid ROS are produced than mitochondria ROS [13].

### 3.5. Detection of ferroptosis

*Cell viability:* Cell Counting Kit-8 and trypan blue assays are well-known methods to detect cell viability and are also appropriate for detecting ferroptosis. Alamar Blue and calcein acetoxymethyl ester viability assays have also been reported to correctly estimate ferroptosis [10].

*Iron level:* Phen Green SK probes are green permeability dyes used to detect intracellular iron in living cells by flow cytometry or confocal microscopy. The green fluorescence of Phen Green SK probes binding to cellular iron is attenuated in ferroptotic cells upon treatment with erastin [53]. Transferrin-bound iron is released at an acidic pH and reduced from ferric to ferrous ions. These ions react with ferrozine to form a violet-colored complex, which is measured spectrophotometrically at 560nm. The absorbance measured at this wavelength is proportional to the serum iron concentration. According to this principle, serum non-heme iron is measured using the Iron/TIBC Reagent designed by Pointe Scientific, Inc. [26]. The tissue non-heme iron levels are measured using the chromogen method [70].

*ROS level:* ROS production can be assayed by flow cytometry using the fluorescent probes C11-BODIPY and 2ʹ,7ʹ-dichlorofluorescin diacetate (DCFH-DA) [10]. The C11-BODIPY probe shifts the fluorescence from red to green in HT-1080 cells upon treatment with erastin. DCFH-DA has no fluorescence and can be hydrolyzed by intracellular esterase to produce DCFH. Intracellular ROS oxidizes non-fluorescent DCFH into green fluorescent DCF and are thus monitored by fluorescence microscopy or flow cytometry. Moreover, dihydrorhodamine enters the cytoplasm and is oxidized into 1,2,3-rhodamine, which can emit fluorescence after entering the cell. The corresponding FITC fluorescence intensity can be detected by flow cytometry after a certain period of accumulation, to detect ROS levels [71]. 4-Hydroxynonenal and malondialdehyde (MDA) are endogenous products derived from the peroxidation of ω-6 PUFAs, which can be used to detect ROS production. MDA and 4-hydroxynonenal in cells, tissues, and serum can be detected using the thiobarbituric acid colorimetric method and enzyme linked immunosorbent assay [26, 72].

*Biomarker protein:* Prostaglandin-endoperoxide synthase-2 (PTGS2), known as cyclooxygenase (COX-2), is upregulated in BJeLR cells upon treatment with either erastin or RSL3. Knockdown of GPX4 also increases PTGS2 mRNA abundance. However, ferroptosis induced by erastin or RSL3 is not affected by COX-2 inhibitor indomethacin treatment, suggesting that PTGS2 does not regulate ferroptosis, but that the increase of PTGS2 could be a marker for ferroptosis [1]. COX-2, ACSL4, and NOX1 are upregulated during ferroptosis, and the expression of GPX4, FTH1, and SLC7A11 proteins is downregulated in ferroptotic cells, thus constituting potential marker proteins for ferroptosis [1, 10, 37, 44, 73]. Furthermore, metallothionein-1 has been identified as a potential biomarker, since it reflects the redox metabolism of sorafenib-induced ferroptosis in cancer cells [74].

### 3.6. Ferroptosis and cardiovascular diseases

Cardiovascular diseases are caused by vascular abnormalities based on atherosclerosis. Hypertension, dyslipidemia, diabetes, and other diseases increase the risk of cardiovascular diseases. There is no direct evidence that ferroptosis is involved in the pathogenesis of atherosclerosis, but relevant studies show that the formation of arterial plaques is related to lipid peroxidation of vascular endothelial cells, iron deposition, lipid deposition, and genesis of micrangium after injury. Studies have revealed that GPX4 overexpression alleviates atherosclerotic lesions in the aortic sinus and aortic tree of ApoE-deficient mice [75]. Furthermore, GPX4 knockdown causes an increase in the levels of lipid peroxidation and induces cytotoxicity. On the other hand, antioxidant vitamin E mitigates lipid peroxidation and cytotoxicity, and delays cell death induced by GPX4 knockdown [75].

Platelet aggregation and thrombus formation are the main causes of myocardial infarction. Markus Wortmann. et al. found that one possible reason for the thromboembolic diseases in tamoxifen-inducible endothelial-specific GPX4 knockout (GPX4^iECKO^) mice might be endothelial cell death, because dying endothelial cells are known to be procoagulant. GPX4^iECKO^ mice display an elevated resting arteriolar vessel tone, which indicates a higher mean arterial blood pressure. MDA, a ferroptosis marker, is elevated in the serum of vitamin E-depleted GPX4^iECKO^ mice. The deficiency of GPX4 combined with deprivation of vitamin E decreases endothelial vasodilators and functionality, which brings about thromboembolic events [76]. It is a remarkable fact that supplementation of vitamin E lowers the risk of venous thromboembolism, especially in women with an anamnesis of venous thromboembolism or family history [77].

The initial manifestation of cardiovascular diseases is a vascular abnormality. Heart failure is the late stage of pathogenesis, and the death of differentiated cardiomyocytes is one of the root causes of cardiovascular diseases. For a long time, caspase-dependent apoptosis was considered the main form of myocardial cell death, but recently ferroptosis has been shown to play an important role in the development of cardiovascular diseases.

Myocardial infarction causes myocardial cell death, ventricular remodeling, and heart failure. The mechanistic target of rapamycin (mTOR) promotes the expression of TfR1 and ferroportin and alters cellular iron flux to protect the heart against pathological stimuli such as ischemia [73, 78]. ROS production is significantly lower in mTOR transgenic cardiomyocytes than in control cardiomyocytes. Overexpression of mTOR regulates ROS and iron metabolism to inhibit ferroptosis in cardiomyocytes, while mTOR deficiency promotes iron-induced ferroptosis [78].

**Figure 5.**
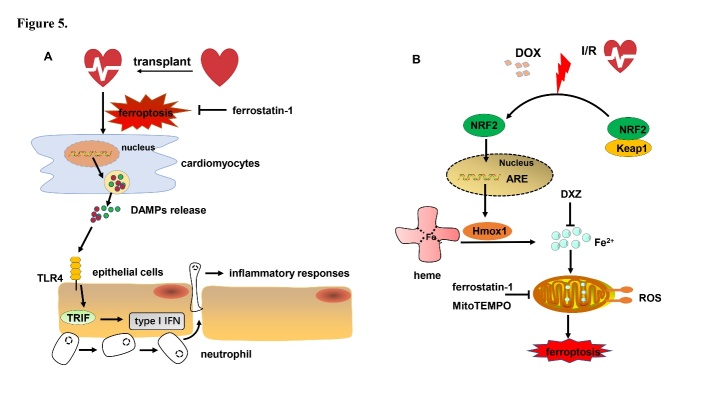
The role of ferroptosis in CVDs. (A) Heart transplantation leads to cardiomyocytes ferroptosis and the release of multiple DAMPs. These DAMPs trigger TLR4/TRIF/Type I IFN signaling in endothelial cells, which recruits neutrophils and results in inflammatory responses. (B) Cardiomyocyte ferroptosis responses to DOX and IRI exposure. Heart exposed to DOX and transient IRI causes Hmox1 upregulation modulated by the Keap1/NRF2 signal pathway. Hmox1 catalyzes heme degradation and causes iron overload, which results in lipid peroxidation and tissue damage. DAMPs: damage associated molecular patterns; TLR4: toll like receptor 4; TRIF: toll-like receptor adaptor molecule 1; DOX: doxorubicin; I/R: ischemia and reperfusion; Keap1: Kelch-like ECH-associated protein 1; IFN: interferon; NRF2: nuclear factor erythroid 2-related factor 2; DXZ: dexrazoxane; ROS: reactive oxygen species; ARE: antioxidant response element.

Heart ischemia and reperfusion induce subsequent cardiac myocyte death, a more common form of damage to the heart muscle. Recent studies have demonstrated that deferoxamine alleviates myocardial IRI and erastin-induced ferroptosis in cardiomyocytes. Rat hearts subjected to IRI or myocardial hypoxia/reoxygenation showed an increase of TfR1 expression and intracellular iron content, accompanied by upregulation of the ferroptosis marker protein ACSL4; this indicates that ferroptosis may participate in IRI or hypoxia/reoxygenation myocardial cell death [25]. Heart transplantation is accompanied by ischemia and reperfusion injury, which causes significant initiation of inflammatory responses. Neutrophils that are recruited to the site of sterile inflammation are triggered by damage-associated molecular patterns generated by ferroptotic cardiomyocytes and induce graft dysfunction [79]. Ferroptosis encourages neutrophils to adhere to damaged blood vessel through a TLR4/TRIF/type I IFN signaling pathway that induces inflammatory responses. Ferrostatin-1 reduces levels of ferroptotic hydroperoxy-arachidonoyl-phosphatidylethanolamine, alleviates cardiomyocyte cell death, reduces left ventricle remodeling and infarcted area, improves left ventricular systolic function, and blocks neutrophil recruitment following heart transplantation (Fig. 5A). Inhibition of ferroptosis protects the heart after heart transplantation [80].

Heart failure is a syndrome in which the pumping function of the heart is impaired, and the cardiac output cannot meet the basic metabolic needs of the whole body. One of the most significant pathophysiological features is cardiomyocyte death [81]. Puerarin, an antioxidant reagent, alleviates heart failure in rats with aortic banding and exhibits cardioprotective functions in clinical trials. A recent study revealed that puerarin reduces iron content and increases ROS elimination in the rat heart failure disease model [82]. DOX is a second-generation anthracycline and has highly effective broad-spectrum anti-tumor activity for the treatment of the malignancy of the blood system and of solid tumors. However, dose-dependent heart failure limits its clinical application. Single treatment based on ferrostatin-1 or on the iron chelator dexrazoxane defends cardiomyocytes against heart failure. Moreover, DOX-induced mortality is reduced in receptor interacting serine/threonine kinase 3 knockout (Ripk3^-/-^) mice compared with wild-type littermates, while ferrostatin-1 treatment further extends the survival of DOX-induced cardiomyopathy compared with the vehicle-treated group in Ripk3^-/-^ mice [26]. A low iron diet significantly raises the survival rate and limits severe heart damage under the condition of *in vivo* DOX-induced heart failure. The expression of Hmox1 was upregulated in DOX-treated murine hearts compared with the vehicle-treated group, as revealed by RNA-sequencing. The Kelch-like ECH-associated protein 1 (Keap1)/NRF2 pathway is activated through oxidative stress in an animal model of DOX-induced cardiomyopathy and myocardial IRI. The transcriptional factor NRF2 binds to the upstream promoter region of the antioxidant response element to enhance the expression of several antioxidative genes, including Hmox1. DOX administration induces ferroptosis caused by upregulation of the NRF2/Hmox1 pathway, acceleration of heme degradation, and release of free iron from heme groups (Fig. 5B) [26]. Significant accumulation of free iron and lipid peroxides is more easily observed in mitochondria than in the cytosol of DOX-treated animal hearts [26]. MitoTEMPO, a mitochondria-targeted superoxide dismutase, limits DOX cardiomyopathy, which indicates that oxidative damage to mitochondria is a major mechanism in ferroptosis-induced heart damage [83].

Cardiomyopathy is a progressive heart disorder accompanied by cardiomyocyte death. Previous studies have shown that patients with thalassemia have an increased risk of cardiomyopathy compared to the normal population, which may be related to the increased iron content in these patients caused by multiple blood transfusions. When the iron storage capacity of the liver is exceeded, iron penetrates the heart, leading to heart failure and rhythm disorders. During this process, iron overload causes cell death, while iron chelators prevent iron overload-induced cell death in cardiomyocytes [84]. Recent studies have shown that hemodynamic dysfunctions associated with iron status and protein and lipid heart oxidative stress are more common in the heart of aged rabbits than in adult rabbits [85].

## 4. Conclusions and Perspectives

Substantial steps have been made in the exploration of the ferroptosis mechanism over the past few years. The main causes of ferroptosis are iron metabolism disorder and ROS production. The homeostasis of PUFAs and iron is important for vital processes such as individual growth and development. When such homeostasis falters, GPX4 is inactivated and cytomembrane PUFAs are peroxided by ROS, thus disrupting membrane functionality and inducing ferroptosis [1]. One of the most prominent features of ferroptosis is PUFA or phospholipid peroxidation, which is indicated by COX-2 or MDA upregulation [26]. The accumulation of excessive iron is another feature of ferroptosis [22]: lipid peroxidation and abnormal iron metabolism cannot be observed in other forms of cell death. GPX4 is the core element of ferroptosis execution and controls the balance of the cellular redox state. The study of ferroptosis is still in the early stage and there are many unanswered questions, such as the exact function of iron in ferroptosis, or the mechanism by which lipid ROS induce ferroptosis. Furthermore, it is still possible more signaling pathways may be involved in ferroptosis.

Cardiomyocyte loss results in dramatic damages to cardiac function and ultimately in heart failure, after which adult cardiomyocytes are unable to regenerate. Therefore, preventing and reducing myocardial cell death is key to improving and restoring cardiac function in cardiovascular diseases. However, this phenomenon was only just discovered in relation to cardiovascular diseases; thus, further studies need to focus on the mechanism of ferroptosis in the development and staging of cardiovascular diseases to help developing precise treatments. Moreover, ferroptosis plays a role in the evolution of cancer, neurotoxicity, and liver and kidney diseases [26, 42, 86-88]. Finally, artemisinin, baicalin, and puerarin inhibit ferroptosis; however, the underlying mechanism is still unclear. In conclusion, ferroptosis, a recently described type of cell death, plays an important role in cardiovascular diseases, but its mechanism needs to be further explored; intervention measures that target ferroptosis may be the basis for new therapeutic strategies for many diseases, including cardiovascular diseases.
